# hTERT Extends the Life of Human Fibroblasts without Compromising Type I Interferon Signaling

**DOI:** 10.1371/journal.pone.0058233

**Published:** 2013-03-05

**Authors:** Miles C. Smith, Erica T. Goddard, Mirna Perusina Lanfranca, David J. Davido

**Affiliations:** Department of Molecular Biosciences, University of Kansas, Lawrence, Kansas, United States of America; McMaster University, Canada

## Abstract

Primary cells are often used to study viral replication and host-virus interactions as their antiviral pathways have not been altered or inactivated; however, their use is restricted by their short lifespan. Conventional methods to extend the life of primary cultures typically utilize viral oncogenes. Many of these oncogenes, however, perturb or inactivate cellular antiviral pathways, including the interferon (IFN) response. It has been previously shown that expression of the telomerase reverse transcriptase (*TERT*) gene extends the life of certain cell types. The effect that TERT expression has on the innate antiviral response to RNA- and DNA-containing viruses has not been examined. In the current study, we introduced the human TERT (*hTERT*) gene into a primary human embryonic lung (HEL-299) cell strain, which is known to respond to the type I IFN, IFN-β. We show that the resulting HEL-TERT cell line is capable of replicating beyond 100 population doublings without exhibiting signs of senescence. Treatment with IFN-β resulted in the upregulation of four model IFN stimulated genes (ISGs) in HEL-299 and HEL-TERT cells. Both cell lines supported the replication of herpes simplex virus type 1 (HSV-1) and vesicular stomatitis virus (VSV) and impaired the replication of both viruses upon IFN-β pretreatment. Introduction of the viral oncoprotein, simian virus 40 (SV40) large T-antigen, which is frequently used to immortalize cells, largely negated this effect. Taken together, our data indicate that expression of hTERT does not alter type 1 IFN signaling and/or the growth of two viruses, making this cell line a useful reagent for studying viral replication and virus-cell interactions.

## Introduction

In performing studies that examine cellular immune responses to viral infections, it is often necessary to work with primary cells, as the efficacy of intrinsic and innate immune pathways are frequently diminished in immortalized cells [1]–[4]. One disadvantage of using primary cells is their limited proliferative capacity in cell culture, which is due in part, to the progressive shortening of telomeres [5].

Telomeres are repetitive nucleoprotein structures that serve to cap the ends of chromosomes, facilitating their replication, and prevent their ends from appearing as DNA breaks [6]. Telomeres are maintained by a complex known as telomerase, whose essential core consists of the catalytic subunit telomerase reverse transcriptase (TERT) and the telomerase RNA template component (TERC) [7], [8]. Along with a number of other factors, TERT is loaded onto the 3′ overhang of existing telomeric DNA and utilizes TERC as a template to add repeats of a guanine-rich sequence, 5′TTAGGG3′, in all vertebrates; concordantly, DNA primase and DNA polymerase are recruited to the new telomeric repeats, subsequently synthesizing the complementary 5′ strand [6]. In the absence of active telomerase, erosion of the telomeres occurs with each successive round of replication, resulting in the loss of telomeric (∼100 bps) sequence [9], [10]. Once telomeres are reduced from their normal 15 kb length to ∼4 kb, DNA damage sensors trigger p53- and pRb-dependent mechanisms that result in cellular senescence, inducing a G_1_ cell cycle arrest [11].

Replicative senescence is thought to be a mechanism of cellular lifespan regulation, preventing diseases such as cancer, and is intrinsic to the health of an organism [12]–[15]. However, for technical reasons it can be desirable to extend the proliferative capacity and prevent the senescence of a primary cell culture or strain. One way to avoid or reverse replicative senescence is transformation with viral oncogenes, such as the simian virus 40 (SV40) large T antigen (TAg) or the human papillomavirus (HPV) E6 and E7 proteins [16]–[18]. In both cases, these viral proteins reverse senescence through the inactivation of p53 and/or pRb. While this allows cells to resume progression through the cell cycle and replicate, these cells still undergo telomeric erosion and ultimately undergo a phenomenon termed crisis [16], where massive cell death occurs due to gross genomic rearrangements and instability in the absence of telomeres. While the estimated 1 in 10^7^ cells (for human cells) that survive crisis exit immortalized [19], [20], this transformation results in the dysregulation of several cellular pathways, including the antiviral type I interferon (IFN) response [1].

The IFN response is an innate antiviral pathway that, upon detection of viral molecular patterns, results in the production and release of the cytokine and type 1 IFN, IFN-β [21]–[23]. IFN-β binds to its cognate receptor in both an autocrine and paracrine manner, activating a signal transduction cascade that ultimately upregulates numerous interferon-stimulated genes (ISGs), which function to limit viral replication. The IFN response serves as a major restriction point for many viruses as evidenced by the increased pathogenesis of these viruses in animal models in which either the type I IFN receptor, IFNAR, or a key signaling molecule, STAT1, are deleted [24]–[30]. One example is herpes simplex virus type-1 (HSV-1), a large double-stranded DNA-containing virus that is estimated to infect 70–90% of adults [31]. Notably, HSV-1 encodes for viral proteins that inactivate or delay this IFN response [32], [33]. Studies examining how HSV-1 counteracts the IFN response are often performed in primary cultures or cell strains, such as human embryonic lung (HEL) cells, because these cells possess a robust IFN response and phenotypes that are apparent in HEL cells are often greatly diminished in transformed lines [1], [34]. A potential drawback with using HEL cells is their rapid progression into senescence.

As part of their differentiation program, human cells cease expressing hTERT, while continuing to produce other essential telomerase subunits such as TERC [35]. It has been shown by a number of labs that the lifespan of fibroblasts is efficiently extended by the reintroduction of hTERT into these cells [36], [37]. Exogenous expression of hTERT presumably allows terminally differentiated fibroblasts to resume the extension of their telomeres, delaying or avoiding the production of signals that trigger replicative senescence and in turn prevents the chromosomal damage encountered by replication through crisis [38]. Unlike transformation with viral oncogenes, fibroblasts that exogenously express hTERT do not, for the most part, exhibit an oncogenic phenotype [39]. Notably, the effect that life-extension by exogenous expression of hTERT on innate antiviral pathways, and in particular the IFN response, has not been examined.

Here we report the creation of a life-extended HEL cell line via transduction of a human diploid primary-like cell strain, HEL-299, with a retrovirus encoding *hTERT*. HEL-299s were chosen as a parental cell line since they are both capable of supporting high levels of HSV-1 and VSV replication and retain a strong innate immune restriction of viral replication [40], [41]. Our results show that the derivative cell line, HEL-TERT, unlike the parental cells, replicated to at least 100 population doublings, exhibited telomerase activity, and failed to undergo either replicative senescence or crisis. Morphologically, HEL-TERT cells appeared indistinguishable from HEL-299 cells. HEL-TERTs responded to IFN-β by upregulating representative ISGs and supported the replication of HSV-1 and VSV to similar levels as HEL-299 cells. Additionally, the introduction of the SV40 large TAg counteracted the IFN-β-directed restriction of HSV-1 and VSV replication. In summary, our data indicate that hTERT extends the replicative potential of human fibroblasts while not perturbing the type 1 IFN response, making these cells a valuable tool in virological and virus-cell interaction studies.

## Materials and Methods

### Cells and Viruses

HEL-299 cells from the American Type Culture Collection (CCL-137), HEL telomerase life-extended (HEL-TERT), and HEL-TERT SV40 large TAg transformed (HEL-TERT-T) cells (the latter two of which were created as part of this work, as detailed below) were maintained in Minimum Essential Medium Eagle Alpha Modification (αMEM) containing 10% fetal bovine serum (FBS), 2 mM L-glutamine, 10 U/mL penicillin, and 10 U/mL streptomycin. In addition, HEL-TERT cells were kept under drug selection using hygromycin-B (Sigma) at 50 µg/mL while HEL-TERT-T cells were maintained under selection with hygromycin-B at 50 µg/mL and phleomycin at 10 µg/mL. HeLa, GP2-293, Vero, and L7 (Vero cells that contain the *ICP0* gene [42]) cells were maintained in Dulbecco’s modified Eagle’s medium (DMEM) containing 5% FBS, 2 mM L-glutamine, 10 U/mL penicillin, and 10 U/mL streptomycin.

HEL-299 (passage 4) cells were transduced with the retroviral vector, pMX-hTERT-hygro vector. pMX-hTERT-hygro was created by subcloning the *hTERT* (catalytic subunit of human telomerase) and hygromycin resistance genes from the vector, pBABE-hygro-hTERT [43] (Addgene plasmid 1773), into the retroviral vector, pMX-GFP [44]. A control vector, pMX-dTERT-hygro, was created by excising a BamHII fragment, which removes the N-terminal 849 residues of hTERT (Uniprot: O14746) [45] (including the TERC-interaction and most of the reverse-transcriptase domains), from pMX-hTERT-hygro. Retroviral stocks were generated using the Pantropic Retroviral Expression System (Clontech) as recommended by the manufacturer. HEL-299 cells were transduced with filtered retroviral stocks and two days later placed under selection with hygromycin B at 100 µg/mL, which was lowered to 50 μg/mL 7 days later for subsequent culturing. HEL-TERT SV40 large TAg-expressing cells were created by transduction with the vector, pLVX-LgT-zeo. pLVX-LgT-zeo was created by subcloning the CMV promoter, SV40 TAg ORF, SV40 early promoter, and zeomycin resistance genes from pBABE-zeo largeTcDNA [46] (Addgene plasmid 1779) into the lentiviral vector, pLVX-AcGFP-N1 (Clontech), replacing the region containing the CMV promoter, AcGFP ORF, phosphoglycerate kinase promoter, and puromycin resistance genes. Lentiviral stocks were prepared essentially as described above for pMX-hTERT-hygro with the inclusion of the lentiviral packaging vector, psPAX2 (Addgene plasmid 12260) during lentiviral stock preparation. HEL-TERT cells were transduced with filtered lentiviral stocks and two days later placed under selection with phleomycin (Invivogen) at 20 µg/mL for 42 days, which was lowered to 10 µg/mL for long term culturing.

KOS was the wild type strain of HSV-1 used in our viral experiments [47]. 7134 is an ICP0-null mutant HSV-1 strain in which the *E. coli lacZ* gene has replaced the *ICP0* open reading frame [48]. KOS and 7134 were grown on Vero cells and titered on Vero or L7 cells, respectively [49], [50]. The vesicular stomatitis virus recombinant, VSV-eGFP, contains the enhanced green fluorescent protein gene between the G and L genes [51] and was a gift from Dr. Asit Pattnaik. VSV-eGFP stocks were grown and titered on Vero cells. Sendai virus (SeV, Cantrell strain) was purchased from Charles River Labratories.

### β-galactosidase Staining

To detect senescence, HEL-299, and moderate and high passage HEL-TERT cells were plated at 1×10^5^ cells per well in 12 well plates and grown to confluence. The cells were fixed in 3.7% formaldehyde, washed twice with 1× phosphate buffered saline (PBS), and stained for β-galactosidase activity as previously described [52]. Cells were viewed with a Nikon Eclipse TE2000-U microscope and photographed with a digital camera (Canon).

### Life-Extension Characterization

Low passage HEL-299 and HEL-TERT cells were plated in 60 mm dishes at 1–2×10^5^ cells per dish. Prior to reaching confluence, the cells were trypsinized, counted with a hemocytometer, and replated at the above-mentioned amount. This was repeated until cells reached senescence and died. Using cell counts and days in culture, the population doublings were determined for each cell line.

### Telomeric Repeat Amplification Protocol (TRAP) Assay

TRAP assays were performed essentially as described [53]. 2×10^5^ HEL-299, HEL-TERT, and HeLa cells were collected, pelleted, and frozen at −80°C. The cell pellets were resuspended in 200 μL of CHAPS lysis buffer (0.5% CHAPS, 10 mM Tris-HCl pH 7.5, 1 mM MgCl_2_, 1 mM EGTA, 3.5% 2-mercaptoethanol, 10% glycerol, 1 mM phenylmethylsulfonyl fluoride, 1 μg/mL aprotinin, and 1 μg/mL leupeptin) and incubated on ice for 30 minutes before cell pellets were collected by centrifugation. Telomeric repeats were amplified in a solution of 10 ng of cell extract, 1× Taq buffer (NEB), 0.2 mM dNTPs, 0.04 μg/μL of T4 Gene 32 Protein (NEB), and 2 U of the Taq polymerase (NEB) containing 0.5 ng/μL of the primers: TS (5′-AATCCGTCGAGCAGAGTT-3′) and ACX (5′-GCGCGG(CTTACC)_3_CTAACC-3′) by polymerase chain reaction (PCR) in an MJ Mini Personal Thermal Cycler (Bio-Rad). Final PCR products were gel electrophoresed on 20% polyacrylamide gel, visualized with ethidium bromide staining, and photographed with a VisiDoc-It Imaging System (UVP).

### Quantitative Reverse Transcriptase Real Time PCR

HEL-299, HEL-TERT, and HEL-TERT-T cells were plated at 1×10^5^ cells per well. Twenty-four hours post-plating, cells were mock treated or treated with human IFN-β at 1000 U/mL (AbD Serotec). At 9 h post treatment, cells were washed twice with PBS and harvested in Trizol (Invitrogen) to isolate total RNA. RNA was converted into cDNA using iScript cDNA synthesis kit (Bio-Rad) according to manufacturers recommendations. For each sample, real time PCR was performed using FastStart SYBR green master (Rox) (Roche) in a StepOnePlus Real-Time PCR System (Applied Biosystems). Transcripts were amplified using the following primer sets: *hTBP* (5′-TGCACAGGAGCCAAGAGTGAA-3′ and 5′-CACATCACAGCTCCCCACCA-3′), *ISG15* (5′-GGTGGACAAATGCGACGAAC-3′ and 5′-ATGCTGGTGGAGGCCCTTAG-3′), *IFIT1* (5′-TAGCCAACATGTCCTCACAGAC-3′ and 5′-GTGCCTTGTAGCAAAGCCCTAT-3′), *IFIT2* (5′-ACGCATTTGAGGTCATCAGGGTG-3′ and 5′-CCAGTCGAGGTTATTTGGATTTGGTT-3′) [54], and *Mx1* (5′-AGAAGGAGCTGGAAGAAG-3′ and 5′-CTGGAGCATGAAGAACTG-3′) [55]. All transcript levels were normalized to hTBP.

### Western Blot

HEL-299, HEL-TERT, and HEL-TERT-T cells were plated at 1.5×10^5^ of cells per well in a 12-well plate. 24 h later, cells were either mock treated or treated with IFN-β at 1000 U/mL for 16 hours before being washed with PBS and then lysed into Red Loading Buffer (62.5 mM Tris-HCl (pH 6.8), 2% SDS, 10% glycerol, 0.01% phenol red, 42 mM DTT) plus with protease inhibitors (1 µg/mL aprotinin, 1 µg/mL leupeptin, 1 mM phenylmethylsulfonyl fluoride). Samples were resolved on a 4–12% Bis-Tris gradient polyacrylamide gel, transferred to nitrocellulose, blocked with 5% BSA in Tris-buffered saline with 0.1% Tween-20 (TBS-T) for 1 h at room temperature. Membranes were probed with an antibody against IFIT1 (PA3-848, Thermo Scientific) diluted in 5% BSA/TBS-T overnight at 4°C. Membranes were washed three times with TBS-T, probed with HRP-conjugated goat-anti-rabbit IgG diluted in 5% BSA/TBS-T for 1 h at room temperature, washed three times with TBS-T, developed with chemiluminescent substrate (Femto ECL, Pierce Laboratories), and detected using an Image Station 4000R (Kodak) and Carestream Molecular Imaging software. The membranes were then striped and probed with β-actin ((I-19)-R, Santa Cruz Biotechnology) as previously described [40]. Images were assembled using Adobe Photoshop and Adobe Illustrator (Adobe Systems).

### Plaque Reduction Assays

Plaque assays for KOS and 7134 on HEL-299, HEL-TERT, and HEL-TERT-T cells (−/+ IFN-β) were carried out as previously described [40]. Images of viral plaques were captured by scanning the immunohistochemically stained plates with a flatbed scanner (Canon).

### HSV-1 Viral Yield Assays

To examine HSV-1 productive infection, HEL-299, HEL-TERT, and HEL-TERT-T cells were plated at 1×10^5^ cells per well in 12 well plates. One day post-plating, cells were mock-treated or treated with 1000 U/mL of human IFN-β. Sixteen hours post-treatment, cells were infected with either KOS or 7134 at 5 plaque forming units (PFU)/cell, washed with PBS (−/+ IFN-β) after 1 hour to remove unabsorbed virus, and placed back in growth medium (−/+ IFN-β). At 24 hours post-infection, cells were harvested and frozen at −80°C. Virally infected samples were thawed and sonicated, and standard plaque assays were performed on either Vero cells (for KOS) or L7 cells (for 7134) to determine viral titers.

### VSV Viral Yield Assays

To measure VSV replication, HEL-299, HEL-TERT, and HEL-TERT-T cells were plated, mock-treated or treated with IFN, and infected as for the HSV-1 yield assays except that cells were infected with VSV-eGFP at 0.1 PFU/cell. At 24 hours post-infection, cells were harvested and frozen at −80°C. Virally infected samples were thawed and sonicated, and standard plaque assays were performed on Vero cells to determine viral titers.

### Antiviral Cytokine-Production Assay

To assess the ability of various cell lines to produce antiviral cytokines, HEL-299, HEL-TERT, or HEL-TERT-T cells were plated at 1×10^5^ in 12-well plates. The next day, the cells were either mock infected with serum-free αMEM or infected with SeV at 100 hemagglutination units (HAU) per 10^6^ cells in serum-free medium for 1 hour, after which the virus was removed from the cells and fresh αMEM containing 10% FCS was added to the cells. Twenty-four hours post infection, SeV-infected HEL cells were irradiated with ultraviolet light to inactive the virus. To test for the production of antiviral cytokines secreted from these cells, duplicate Vero cell monolayers (2×10^5^ cells per well in 12-well plates) were exposed to HEL supernanes. In addition, one set of Vero cells were treated with either fresh αMEM or αMEM containing IFN-β at 1, 10, 100, or 1000 U/mL as positive controls. Six hours later, untreated and treated Vero cells were infected with VSV-eGFP at ∼200 PFU/well. At 1 hour post infection, the Vero cells were overlaid with αMEM containing 10% FCS and 1% methylcellulose. Twenty-four hours post-VSV infection, the methylcellulose was removed, Vero cells were washed with PBS and fixed with 3.7% formaldehyde, and fluorescent plaques were counted.

## Results

### HEL-TERT Cells Exhibit an Expanded Proliferative Capacity

**Figure 1:**
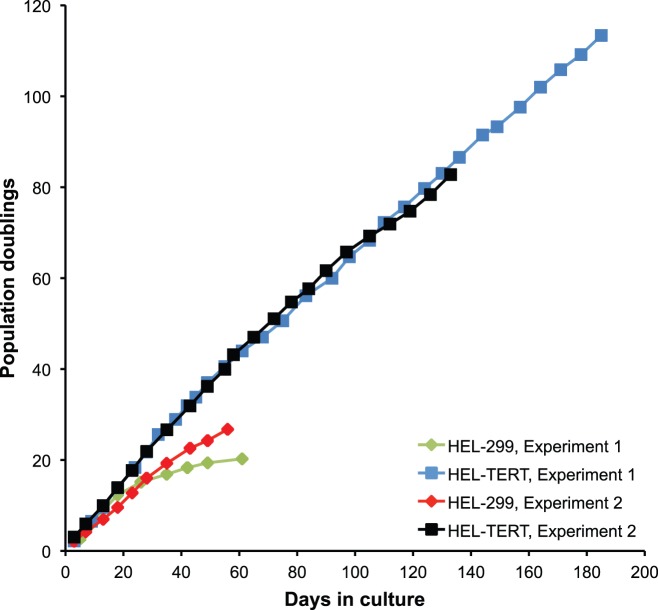
HEL-TERT cells are life-extended compared to HEL-299 cells. HEL-TERT and HEL-299 cells were plated as duplicate cultures in 60 mm dishes at 1×10^5^ and 2×10^5^ cells per plate, respectively. For each passaging, cells were counted and re-plated. Population doublings were determined by using cell counts and days in culture.

HEL-299 cells are a primary strain that have been used to study viral replication and the type 1 IFN response [40], [56], [57]. This cell strain, nonetheless, can only be passaged in culture a limited number of times before undergoing senescence [58]. We wanted to determine whether ectopic expression of hTERT in HEL-299 cells would allow for a longer period of culturing. HEL-299s were transduced with a retrovirus encoding both hTERT and hygromycin resistance. The resulting antibiotic resistant mass population, hereafter called HEL-TERT, were then used in subsequent experiments. To first examine whether hTERT conferred an extended ability to replicate, HEL-299 and HEL-TERT cells were maintained in culture for an extended period of time, comparing the number of population doublings to days in culture. As expected, HEL-299s proliferated just under 60 days in culture and underwent a total of 23.5 population doublings (from two experiments); at which point the cells ceased to divide and underwent widespread cell death two weeks later (Figure 1). It should be noted that in a couple of instances, HEL-299 cells were able to undergo approximately 35 population doublings (data not shown). In contrast, the HEL-TERT cells were maintained in culture for 185 days and went through 114 population doublings (Figure 1), at which point the experiment was terminated. Transduction of HEL-299 cells with a retroviral vector that either expresses the green fluorescent protein or contains a deletion in *hTERT* failed to extend the life span of the HEL-299s (data not shown). These results demonstrate that expression of hTERT significantly extends the life span of HEL-299 cells.

### HEL-TERT Cells Contain Active Telomerase

**Figure 2:**
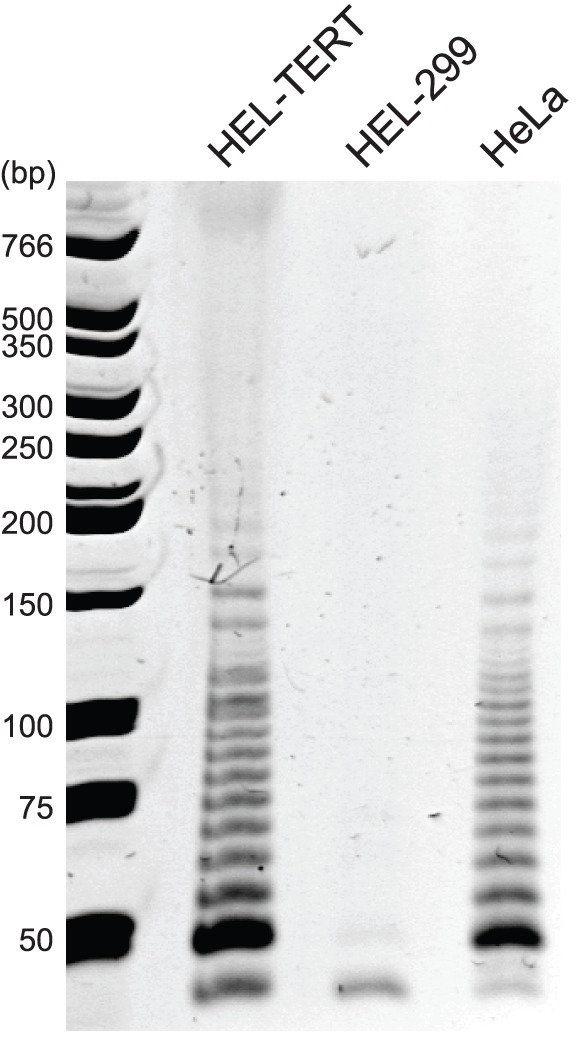
Telomerase activity is detectable in HEL-TERT and HeLa cells but not HEL-299 cells. HEL-299 (3 population doublings), HEL-TERT (3 population doublings), and HeLa cells were examined for telomerase activity using the TRAP assay. HeLa cells were used as a positive control for telomerase activity. The numbers at the left side of the figure are DNA size markers (bp: base pair).

To establish that transduced hTERT resulted in telomerase activity in HEL-TERT cells, we performed TRAP assays. In this assay, telomerase activity is monitored by examining the laddering or amplification of 6 base-pair 5′TTAGGG3′ telomeric repeats [59]. HeLa cells, which express hTERT [60], exhibited the characteristic 6 bp laddering, while the non-immortalized HEL-299 failed to do so (Figure 2). Unlike the parental cell line, the HEL-TERT cells showed a clear laddering effect, indicating that exogenous hTERT is active and capable of extending telomeres. As a control, we determined that our samples did not contain a PCR inhibitor by amplifying the cellular promyelocytic leukemia (*PML*) gene (data not shown). Thus, HEL-TERT cells contain active telomerase, suggesting that the extended proliferative capacity of this cell line can be attributed to the maintenance of telomeres.

### Prolonged Culture of HEL-TERTs does not Result in Senescence

**Figure 3:**
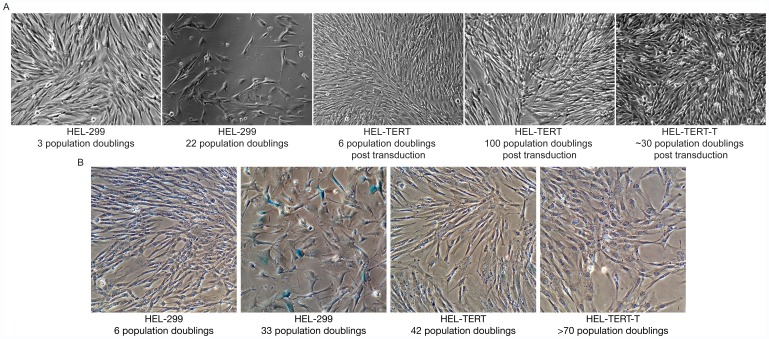
HEL-299, HEL-TERT, and HEL-TERT-T cell morphology and senescence. **A.** Transduction of HEL-299 cells with hTERT does not alter morphology. Light microscopy of live HEL-299 cells at 3 (left panel) and 22 population doublings (left middle panel), HEL-TERT cells after 6 (middle panel) and 100 (right middle panel), and HEL-TERT-T (far right panel) after approximately 30 population doublings. **B.** HEL-TERT cells fail to exhibit at least one sign of senescence. HEL-299 cells at 6 (left panel) and 33 (left middle panel), HEL-TERT cells after 42 (right middle panel), and HEL-TERT-T cells at approximately 70 (right panel) population doublings were stained for β-galactosidase activity.

As fibroblasts reach senescence, they exhibit characteristic changes in cellular morphology, such as an increase in area, due to dysregulation of cytoskeleton elements [5], [61]. Additionally, senescent cells can be detected by their upregulation of a lysosomal β-galactosidase [62]. When we compared low (6 population doublings) and high (20 population doublings) passage HEL-299 cells, we noted that many of the higher passage cells exhibited a clear enlargement of the cytoplasm, with a change from their typical narrow, drawn-out morphology to one that was shortened and/or broader (Figure 3A). When we compared the HEL-TERT cells to low passage HEL-299 cells, we were able to detect little if any morphological changes either shortly after transduction with hTERT or at 100 population doublings later (Figure 3A). As part of these studies, we also examined another HEL-TERT derivative cell line that expresses SV40 large Tag (hereafter named HEL-TERT-T). SV40 large Tag is known to alter the IFN-response [63]. From this experiment, HEL-TERT-T cells appeared to have an altered cellular morphology, with the cells decreasing in length and broadening in width. When we examined all the cell types for senescence-associated β-galactosidase activity, β-galactosidase activity was clearly detected in older HEL-299 cells, whereas we failed to detect β-galactosidase activity in either low passage HEL-299 or low or high passage HEL-TERT, or high passage HEL-TERT-T cells (Figure 3B). These results indicate that not only do HEL-TERT cells retain their ability to proliferate but also fail to exhibit signs of senescence.

### Treatment of HEL-TERT Cells with Human IFN-β Induces Strong ISG Expression

**Figure 4:**
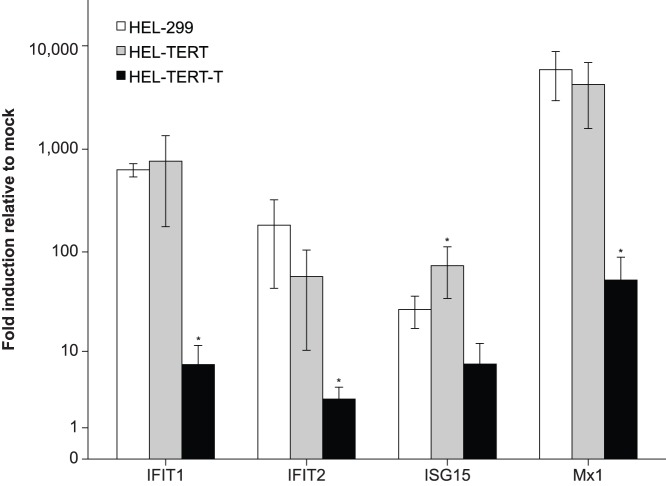
HEL-TERT but not HEL-TERT-T cells show ISG induction at levels similar to HEL-299 cells after IFN stimulation. HEL-299 and HEL-TERT cells were treated or mock treated with 1000 U/mL of human IFN-β. At 9 hours post treatment, total RNA was isolated from cells and reversed transcribed into cDNA for qRT-PCR analysis to monitor *IFIT1*, *IFIT2*, *ISG15*, and *Mx1* transcript levels. Data represents the means of 6 samples; error bars represent the standard errors of the means. *p<0.05, one-way ANOVA, Bonferroni’s multiple comparison post-test, compared to HEL-299 levels.

**Figure 5:**
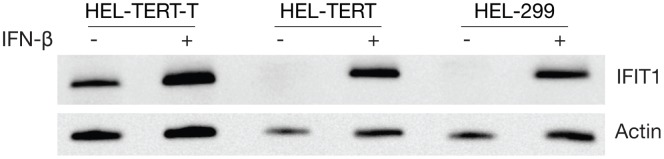
IFIT1 protein production is induced to similar levels by IFN-β in HEL-299 and HEL-TERT cells. HEL-299, HEL-TERT, and HEL-TERT-T cells were mock treated or treated with 1000 U/mL of IFN-β and harvested 9 hours later. Cell lysates were analyzed for IFIT1 or β-actin protein production by western blot.

Because the IFN response has been reported to be altered in immortalized cells [2]–[4], we decided to examine the effect that exogenous hTERT had on ISG levels. HEL-299, HEL-TERT, and HEL-TERT-T cells were stimulated with IFN-β for 9 h and the transcript levels of four prototypic ISGs (*ISG15*, *IFIT1*, *IFIT2*, and *Mx1*) were monitored by qRT-PCR. Both HEL-299 and HEL-TERT cells showed robust upregulation in the transcript levels of all four genes after the addition of IFN-β (Figure 4). Three of the ISGs induced to similar levels between the two cell lines while *ISG15* was induced to slightly higher levels in the HEL-TERTs. On the other hand, the overall upregulation of these genes upon IFN-β treatment was greatly diminished in HEL-TERT-T cells. When we examined IFIT1 protein levels, we found that, as expected [64], unstimulated HEL-299 and HEL-TERT cells contained little to no detectable IFIT1; however, IFIT1 was readily detected 9 hours after IFN-β treatment (Figure 5). Notably, IFN-treated HEL-299 and HEL-TERT cells showed comparable levels of IFIT1 protein. HEL-TERT-T cells, on the other hand, showed persistent production of IFIT1 and a greatly reduced difference between the unstimulated and IFN-treated states (as compared to that found in the other two cell lines), in agreement with a previous report [65]. Thus, the ectopic expression of hTERT in HEL-299 cells via retroviral transduction does not largely affect the ability of HEL cells to induce the expression of these four ISGs by IFN-β nor does it lead to a dysregulation of ISG protein production (i.e., IFIT1) as does expression of TAg.

### HSV-1 and VSV Replicate to Comparable Levels, +/− IFN-β, in HEL-299 and HEL-TERT Cells

**Figure 6:**
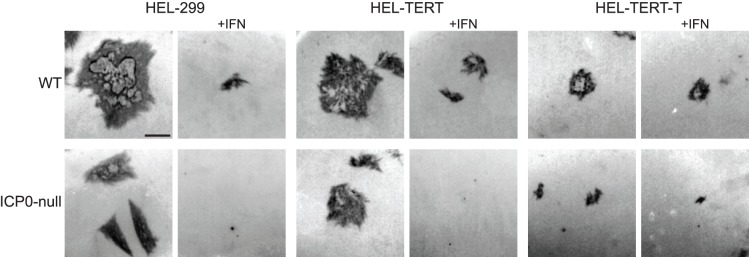
HSV-1 shows similar plaque size and morphology on HEL-299 and HEL-TERT cells. HEL-299, HEL-TERT, and HEL-TERT-T cells were mock or pretreated with IFN-β for 16 h and then infected with WT HSV-1 or an ICP0-null mutant, and plaques for both viruses were visualized by immunohistochemistry three days post-infection. Bar = 1 mm.

**Figure 7:**
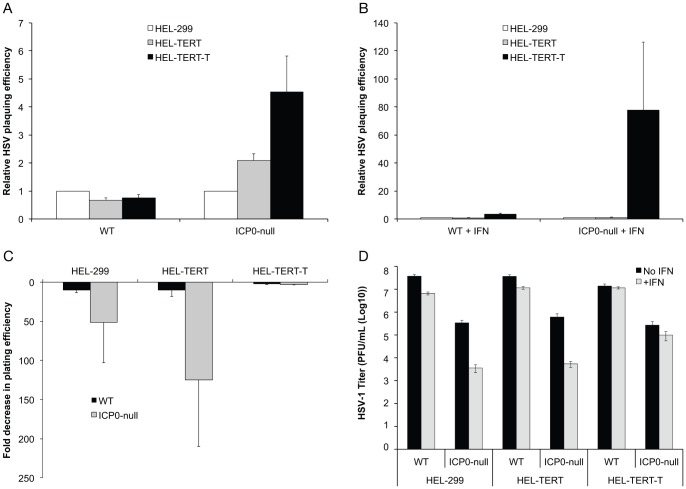
Replication of HSV-1 is diminished by IFN-β in HEL-299 and HEL-TERT but not HEL-TERT-T cells. A and B. HEL-299, HEL-TERT, and HEL-TERT-T cells were mock (A) or pre-treated with IFN-β (1000 U/mL) (B) and were infected 16 h post treatment with 10-fold serially diluted stocks of WT HSV-1 or an ICP0-null mutant. Plaques were visualized by immunohistochemistry 3 days post-infection. An average of three experiments is shown. Data is presented as the ratio of plaques formed on the indicated cell line to that on HEL-299 cells. **C.** Data generated for A and B, but presented as a ratio of the number of plaques formed on mock-treated cells compared to that on IFN-treated cells. **D.** HEL-299, HEL-TERT, and HEL-TERT-T cells were mock or pre-treated with IFN-β (1000 U/mL) and were infected (16 h post treatment) with either WT HSV-1 or the ICP0-null mutant at an MOI of 5 PFU/cell. Samples were harvested 24 h post-infection. Viral titers were determined by plaque assays. An average of three experiments is shown. In all cases, error bars represent the standard errors of the means.

As another measure to assess whether the IFN response is active and functional in HEL-TERT cells, we examined the replication of three viruses in the presence of IFN-β. For these studies, we chose HSV-1, which is largely resistant to type I IFNs, as well as both an ICP0-null mutant of HSV-1 and VSV, as these latter two viruses are sensitive to type I IFNs [66]–[68]. Initially, we examined the ability of wildtype (WT) and ICP0-null HSV-1 to form plaques on untreated and IFN-β-treated HEL-299, HEL-TERT, and HEL-TERT-T cells. Both viruses had visually comparable plaque sizes on both HEL-299 and HEL-TERT cell types (Figure 6), even on higher passage HEL-TERT cells (data not shown). Plaques appeared to be slightly smaller on HEL-TERT-T cells, which is most likely due a decrease in the size of the cells that occurred upon transduction of TAg (Figure 3A). When the cells were pretreated with IFN-β, there was a large decrease in plaque size for both WT HSV-1 and the ICP0-null mutant on HEL-299 and HEL-TERT cells, while plaque size on the HEL-TERT-T cells remained largely the same. The ability of WT virus to form plaques was similar on the three cell lines in untreated cells (Figure 7A), though the ICP0-null virus showed slight increases of 2-fold and 4.5-fold on HEL-TERT and HEL-TERT-T cells, respectively. Upon the addition of IFN-β, the plaquing efficiencies of the WT and ICP0-null viruses were decreased ∼10 fold and ∼50-100-fold, respectively, on both HEL-299 and HEL-TERT cells (Figure 7C). In contrast to the introduction of hTERT into HEL-299 cells, expression of large TAg greatly diminished the ability of IFN-β to restrict the plaquing of either WT or the ICP0-null virus (3-fold for either) (Figure 7C), resulting in a nearly 80-fold increase in the plating efficiency of the ICP0-null virus on IFN-treated HEL-TERT-T cells as compared to IFN-treated HEL-299 cells (Figure 7B). To further examine the effect of hTERT on HSV-1 replication, we also performed viral yield assays in the three cell types. (Figure 7D). WT HSV-1 replicated to comparable levels in all three cell lines, with IFN-pretreatment producing a slight reduction in yields from both HEL-299 and HEL-TERT but not from HEL-TERT-T cells. Like WT HSV-1, the ICP0-null mutant replicated nearly as well among the three cell types in untreated cells; however, IFN-pretreatment resulted in a 100-fold decrease of viral yields in HEL-299 and HEL-TERT cells while producing little to no effect in HEL-TERT-T cells.

**Figure 8:**
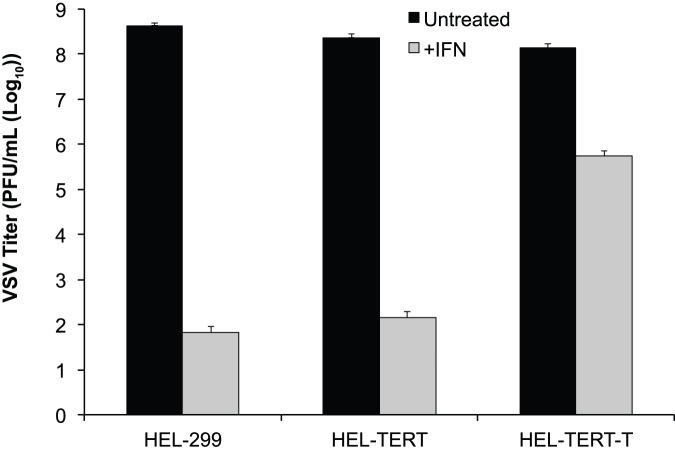
Replication of VSV is diminished by IFN-β in HEL-299 and HEL-TERT but not HEL-TERT-T cells. HEL-299, HEL-TERT, and HEL-TERT-T cells were mock or pre-treated with IFN-β (1000 U/mL) and were infected (16 h post treatment) with VSV-eGFP at an MOI of 0.1 PFU/cell. Samples were harvested 24 h post-infection. Viral titers were determined by plaque assays. An average of three experiments is shown. In all cases, error bars represent the standard errors of the means.

To monitor reductions in VSV production, we again performed viral yield assays in the presence and absence of IFN-β. Just as for HSV-1, VSV grew equally well among the three cell lines in untreated cells. In IFN-β-treated cells, however, VSV growth in both HEL-299 and HEL-TERT cells was reduced by >10^6^-fold while it was reduced by only 200-fold in pretreated HEL-TERT-T cells (Figure 8). Thus, HEL-TERT cells are similar to HEL-299s in their ability to support the replication of two genetically distinct viruses, and they retain an IFN response that is as functional as the parental cell line. Overall, ectopically expressed hTERT does not appear to adversely affect viral replication or the type I IFN response in a human lung fibroblast cell strain.

### Effect of hTERT on Antiviral Cytokine Production

In addition to determining its effect on ISG upregulation and on the efficacy of an IFN-induced antiviral state, we decided to assess whether ectopic hTERT expression altered the ability of cells to produce IFN and other antiviral cytokines in response to infection. We used infection by SeV, which is known to be a strong inducer of IFNs and other antiviral cytokines in human cells [69]. For this assay, media from uninfected and infected HEL cells are placed onto naïve Vero cells, which respond to but cannot produce IFN, and restrictions on VSV plaquing are monitored [70], [71]. Media from mock-infected HEL cells had no effect on the ability of VSV to plaque on Vero cells while IFN-β pretreatment, at the highest level tested (1000 U/mL), was able to reduce the number of plaques formed by approximately 35-fold (Table 1). When we tested the ability of media from SeV-infected HEL cells, we found that media from all three cell types were capable of lowering the number of plaques that formed by 5–7 fold. These reductions were similar to the antiviral activity of 100 U/mL of IFN-β. Although we are unable to distinguish between IFN-β or among the IFN-α subtypes with this assay, the protective effect produced by media from HEL-299 and HEL-TERT cells was identical and suggests that hTERT does not affect the activation of IFN-production in response to viral infection.

**Table 1:** Ectopic hTERT expression does not affect the ability of HEL cells to produce antiviral cytokines.

	Treatment	Number of plaqueson Vero cells
HEL-299	Mock	224.0±19.4
	SeV	40.6±4.44
HEL-TERT	Mock	234.8±19.3
	SeV	35.0±6.54
HEL-TERT-T	Mock	219.6±14.7
	Sev	28.6±7.60
IFN-β		
	Untreated	215.0±5.0
	1 U/mL	227.5±3.5
	10 U/mL	200.5±9.5
	100 U/mL	27.5±0.50
	1000 U/mL	6.00±2.0

HEL-299, HEL-TERT, and HEL-TERT-T cells were mock- or SeV-infected at 100 HAU/10^6^ cell. At 24 hpi, the media were transferred to naïve Vero cell monolayers. 6 h later, the Vero cells were infected with ∼200 PFU of VSV-eGFP per well. At 24 hpi with VSV-eGFP, the Vero cells were fixed and the number of fluorescent plaques counted. Data shown represents the average of two experiments performed in duplicate (± standard errors of the means).

## Discussion

Due to their unperturbed DNA damage, senescence, and antiviral pathways, primary cells are considered biologically relevant cells when studying how these cellular processes affect viral replication. However, their limited ability to proliferate makes their use in examining these pathways technically challenging. For example, the establishment of a cell line depleted for a particular cellular protein is generally difficult to generate because of the rapid and inevitable onset of senescence. Thus, in studying cell-virus interactions, there is a need for life-extended cell lines that retain many of the characteristics of a primary cell (e.g., antiviral responses) while allowing for the analysis of specific cellular genes or proteins (e.g., depletion, gene knockout). It is possible to immortalize primary cells with viral and cellular oncoproteins, but immortalization can result in alterations of cellular processes and inhibit antiviral pathways, affecting the replication of wild-type and mutant viruses [72]–[75]. Another approach is to use the *TERT* gene, which has been reported to extend the life of human fibroblasts [36], and avoids many of the problems associated with cellular or viral oncogene immortalization or transformation [39]. Prior to this study, the effect of hTERT expression on the IFN response had not, to the best of our knowledge, been examined.

The traditional approach used to immortalize primary cells has been the introduction of cellular or viral oncogenes. The most commonly used of these include E1A and E1B from adenovirus [76], E6 and E7 from human papillomavirus [17], and large TAg from SV40 [20], [77]. In general, these proteins bypass senescence by the inactivation of one or both of the tumor suppressor proteins, p53 and pRb [78]–[80]. Unfortunately, in addition to perturbing the cell cycle, many of these viral proteins also serve to antagonize or inactivate antiviral pathways in order to promote viral replication. E1A, E6, E7, and large TAg are capable of disrupting the activity of, among others, cellular histone deacetylases [81] and CBP/p300 [82]–[84] resulting in widespread transcriptional and epigenetic changes [85]–[89]. In the case of E1A, this interaction prevents the major type I IFN transcription factor, STAT1, from binding to CBP/p300 and upregulating ISGs [90]. Likewise, E6 is capable of preventing activation of the IFN response by blocking the transcriptional activity of IRF3 [91]. E1B proteins inhibit apoptosis and are capable of inactivating the cellular DNA damage response [92]–[94]. Large TAg, on the other hand, has recently been reported to activate STAT1 and induce ISGs upregulation in the absence of IFN-exposure [65]. In agreement with this, we saw persistent production of IFIT1 protein in TAg-transduced cells. However, the expression of large TAg, independent of SV40 infection, has also been shown to decrease the phosphorylation of the cellular translation factor eIF2α by an IFN effector, double-stranded RNA protein kinase (PKR) [95]. This decrease in phosphorylation increases the translation capability of viral mRNAs. Similarly, our results show that while large TAg may lead to high levels of ISG protein production, it functionally inactivates the IFN response. Furthermore, immortalization by mechanisms not involving viral oncogenes may inactivate antiviral pathways, as observed with the loss of induction of ISGs in immortalized cells derived from Li-Fraumeni patients [1].

Our approach in this study was to extend the life of human fibroblasts with hTERT. Cells transformed with hTERT arrest in response to serum starvation, maintain anchorage dependence, double at a rate similar to untransformed cells, and do not exhibit genomic instability [39]. While it has been reported that expression of hTERT can alter the expression of a limited number of genes, none of these have an apparent role in antiviral pathways [96]. We found that hTERT expression does not interfere with the upregulation of four representative ISGs (*ISG15*, *IFIT1*, *IFIT2*, and *Mx1*), does not lead to aberrant ISG protein production, nor does its expression affect the replication of two genetically distinct viruses, HSV-1 and VSV. This is in agreement with previous work demonstrating that exogenous expression of hTERT in fibroblasts does not affect the replication of human cytomegalovirus [97], [98] nor does it affect the upregulation of the IFN-induced senescence mediator, IFI16, upon IFN-stimulation [99]. Furthermore, unlike SV40 large TAg, exogenous hTERT did not impair the ability of IFN to restrict the replication of VSV or an ICP0-null mutant of HSV-1, both viruses being quite sensitive to the effects of IFN-β, nor did it hinder the ability of HEL cells to produce antiviral cytokines in response to viral infection. While we did observe slight differences in the levels of induction for the four ISGs between HEL-299 and HEL-TERT cells, these differences failed to translate into an appreciable effect on the ability of IFN-β to suppress replication of VSV or the ICP0-null HSV-1 mutant. hTERT overexpression has been reported to enhance the formation of apoptotic markers during HSV-1 infection in HeLa cells, which express the human papillomavirus E6 and E7 oncoproteins, and sensitizes them to apoptosis [100]. Our results, however, suggest that exogenous expression of hTERT in a primary cell strain has little impact on viral replication.

In conclusion, HEL-TERTs are permissive for HSV-1 and VSV growth, have a robust antiviral response, and a significantly enhanced lifespan. They recapitulate the phenotype of an HSV-1 ICP0-mutant, which is known to be complemented by the loss of proteins involved in the DNA damage response [101], antiviral pathways [102], or overexpression of certain cyclins [103], suggesting that these pathways are unperturbed. Because the phenotypes of certain HSV-1 mutants are only apparent in primary cells, we believe the HEL-TERT cell line to be an ideal choice due to their longevity and robust antiviral response. Additionally, they will allow for the establishment of derivative cell lines that are depleted or overexpress targets of interest, facilitating a better understanding of cellular pathways (including the IFN response) and the viruses that alter these pathways.

## References

[1] Fridman AL, Tang L, Kulaeva OI, Ye B, Li Q, et al (2006) Expression profiling identifies three pathways altered in cellular immortalization: interferon, cell cycle, and cytoskeleton. J Gerontol A Biol Sci Med Sci 61: 879–889. 16960018 10.1093/gerona/61.9.879

[2] Kulaeva OI, Draghici S, Tang L, Kraniak JM, Land SJ, et al (2003) Epigenetic silencing of multiple interferon pathway genes after cellular immortalization. Oncogene 22: 4118–4127 doi:10.1038/sj.onc.1206594. 12821946 10.1038/sj.onc.1206594

[3] Shou J, Soriano R, Hayward SW, Cunha GR, Williams PM, et al (2002) Expression Profiling of a Human Cell Line Model of Prostatic Cancer Reveals a Direct Involvement of Interferon Signaling in Prostate Tumor Progression. PNAS 99: 2830–2835 doi:10.1073/pnas.052705299. 11880635 10.1073/pnas.052705299 PMC122433

[4] Untergasser G, Koch HB, Menssen A, Hermeking H (2002) Characterization of Epithelial Senescence by Serial Analysis of Gene Expression Identification of Genes Potentially Involved in Prostate Cancer. Cancer Res 62: 6255–6262. 12414655

[5] Hayflick L (1965) The limited in vitro lifetime of human diploid cell strains. Experimental Cell Research 37: 614–636 doi:10.1016/0014-4827(65)90211-9. 14315085 10.1016/0014-4827(65)90211-9

[6] Gilson E, Geli V (2007) How telomeres are replicated. Nat Rev Mol Cell Biol 8: 825–838 doi:10.1038/nrm2259. 17885666 10.1038/nrm2259

[7] Blackburn EH, Greider CW, Henderson E, Lee MS, Shampay J, et al (1989) Recognition and elongation of telomeres by telomerase. Genome 31: 553–560. 2698831 10.1139/g89-104

[8] Feng J, Funk WD, Wang S-S, Weinrich SL, Avilion AA, et al (1995) The RNA Component of Human Telomerase. Science 269: 1236–1241 doi:10.1126/science.7544491. 7544491 10.1126/science.7544491

[9] Zhao Y, Hoshiyama H, Shay JW, Wright WE (2008) Quantitative Telomeric Overhang Determination Using a Double-Strand Specific Nuclease. Nucl Acids Res 36: e14 doi:10.1093/nar/gkm1063. 18073199 10.1093/nar/gkm1063 PMC2241892

[10] Harley CB, Futcher AB, Greider CW (1990) Telomeres shorten during ageing of human fibroblasts., Published online: 31 May 1990; | doi:101038/345458a0 345: 458–460. doi:10.1038/345458a0. 10.1038/345458a0 2342578

[11] Neumeister P, Albanese C, Balent B, Greally J, Pestell RG (2002) Senescence and epigenetic dysregulation in cancer. The International Journal of Biochemistry & Cell Biology 34: 1475–1490 doi:10.1016/S1357-2725(02)00079-1. 12200040 10.1016/s1357-2725(02)00079-1

[12] Campisi J, D’ Adda di Fagagna F (2007) Cellular senescence: when bad things happen to good cells. Nat Rev Mol Cell Biol 8: 729–740 doi:10.1038/nrm2233. 17667954 10.1038/nrm2233

[13] Campisi J (2001) Cellular senescence as a tumor-suppressor mechanism. Trends in Cell Biology 11: S27–S31 doi:10.1016/S0962-8924(01)02151-1. 11684439 10.1016/s0962-8924(01)02151-1

[14] Braig M, Schmitt CA (2006) Oncogene-Induced Senescence: Putting the Brakes on Tumor Development. Cancer Res 66: 2881–2884 doi:10.1158/0008-5472.CAN-05-4006. 16540631 10.1158/0008-5472.CAN-05-4006

[15] Smith JR, Pereira-Smith OM (1996) Replicative Senescence: Implications for in Vivo Aging and Tumor Suppression. Science 273: 63–67 doi:10.1126/science.273.5271.63. 8658197 10.1126/science.273.5271.63

[16] Beausejour CM, Krtolica A, Galimi F, Narita M, Lowe SW, et al (2003) Reversal of human cellular senescence: roles of the p53 and p16 pathways. EMBO J 22: 4212–4222 doi:10.1093/emboj/cdg417. 12912919 10.1093/emboj/cdg417 PMC175806

[17] Hawley-Nelson P, Vousden KH, Hubbert NL, Lowy DR, Schiller JT (1989) HPV16 E6 and E7 proteins cooperate to immortalize human foreskin keratinocytes. The EMBO Journal 8: 3905. 2555178 10.1002/j.1460-2075.1989.tb08570.x PMC402081

[18] Hudson JB, Bedell MA, McCance DJ, Laiminis LA (1990) Immortalization and Altered Differentiation of Human Keratinocytes in Vitro by the E6 and E7 Open Reading Frames of Human Papillomavirus Type 18. J Virol 64: 519–526. 2153221 10.1128/jvi.64.2.519-526.1990 PMC249139

[19] Shay JW, Wright WE (1989) Quantitation of the frequency of immortalization of normal human diploid fibroblasts by SV40 large T-antigen. Experimental Cell Research 184: 109–118 doi:10.1016/0014-4827(89)90369-8. 2551703 10.1016/0014-4827(89)90369-8

[20] Shay JW, Van Der Haegen BA, Ying Y, Wright WE (1993) The frequency of immortalization of human fibroblasts and mammary epithelial cells transfected with SV40 large T-antigen. Exp Cell Res 209: 45–52 doi:10.1006/excr.1993.1283. 8224005 10.1006/excr.1993.1283

[21] Randall RE, Goodbourn S (2008) Interferons and viruses: an interplay between induction, signalling, antiviral responses and virus countermeasures. J Gen Virol 89: 1–47 doi:10.1099/vir.0.83391-0. 18089727 10.1099/vir.0.83391-0

[22] Stark GR, Kerr IM, Williams BRG, Silverman RH, Schreiber RD (1998) How Cells Respond to Interferons. Annual Review of Biochemistry 67: 227–264 doi:10.1146/annurev.biochem.67.1.227. 10.1146/annurev.biochem.67.1.227 9759489

[23] Katze MG, He Y, Gale M (2002) Viruses and interferon: a fight for supremacy. Nat Rev Immunol 2: 675–687 doi:10.1038/nri888. 12209136 10.1038/nri888

[24] Conrady CD, Halford WP, Carr DJJ (2011) Loss of the Type I Interferon Pathway Increases Vulnerability of Mice to Genital Herpes Simplex Virus 2 Infection. J Virol 85: 1625–1633 doi:10.1128/JVI.01715-10. 21147921 10.1128/JVI.01715-10 PMC3028887

[25] Van den Broek MF, Müller U, Huang S, Aguet M, Zinkernagel RM (1995) Antiviral defense in mice lacking both alpha/beta and gamma interferon receptors. Journal of virology 69: 4792–4796. 7609046 10.1128/jvi.69.8.4792-4796.1995 PMC189290

[26] Hwang SY, Hertzog PJ, Holland KA, Sumarsono SH, Tymms MJ, et al (1995) A null mutation in the gene encoding a type I interferon receptor component eliminates antiproliferative and antiviral responses to interferons alpha and beta and alters macrophage responses. Proceedings of the National Academy of Sciences 92: 11284. 10.1073/pnas.92.24.11284 PMC40616 7479980

[27] Muller U, Steinhoff U, Reis LF, Hemmi S, Pavlovic J, et al (1994) Functional Role of Type I and Type II Interferons in Antiviral Defense. Science 264: 1918–1921 doi:10.1126/science.8009221. 8009221 10.1126/science.8009221

[28] Bereczky S, Lindegren G, Karlberg H, Åkerström S, Klingström J, et al (2010) Crimean–Congo Hemorrhagic Fever Virus Infection Is Lethal for Adult Type I Interferon Receptor-Knockout Mice. J Gen Virol 91: 1473–1477 doi:10.1099/vir.0.019034-0. 20164263 10.1099/vir.0.019034-0

[29] Detje CN, Meyer T, Schmidt H, Kreuz D, Rose JK, et al (2009) Local Type I IFN Receptor Signaling Protects Against Virus Spread Within the Central Nervous System. J Immunol 182: 2297–2304 doi:10.4049/jimmunol.0800596. 19201884 10.4049/jimmunol.0800596

[30] Durbin JE, Hackenmiller R, Simon MC, Levy DE (1996) Targeted Disruption of the Mouse Stat1 Gene Results in Compromised Innate Immunity to Viral Disease. Cell 84: 443–450 doi:10.1016/S0092-8674(00)81289-1. 8608598 10.1016/s0092-8674(00)81289-1

[31] Fields BN, Knipe DM, Howley PM, Griffin DE (2007) Fields Virology. Lippincott Williams & Wilkins. 1650 p.

[32] Smith MC, Boutell C, Davido DJ (2011) HSV-1 ICP0: paving the way for viral replication. Future Virology 6: 421–429 doi:10.2217/fvl.11.24. 21765858 10.2217/fvl.11.24 PMC3133933

[33] Paladino P, Mossman KL (2009) Mechanisms employed by herpes simplex virus 1 to inhibit the interferon response. J Interferon Cytokine Res 29: 599–607 doi:10.1089/jir.2009.0074. 19694546 10.1089/jir.2009.0074

[34] Paladino P, Collins SE, Mossman KL (2010) Cellular Localization of the Herpes Simplex Virus ICP0 Protein Dictates Its Ability to Block IRF3-Mediated Innate Immune Responses. PLoS ONE 5: e10428 doi:10.1371/journal.pone.0010428. 20454685 10.1371/journal.pone.0010428 PMC2861674

[35] Meyerson M, Counter CM, Eaton EN, Ellisen LW, Steiner P, et al (1997) hEST2, the Putative Human Telomerase Catalytic Subunit Gene, Is Up-Regulated in Tumor Cells and during Immortalization. Cell 90: 785–795 doi:10.1016/S0092-8674(00)80538-3. 9288757 10.1016/s0092-8674(00)80538-3

[36] Bodnar AG, Ouellette M, Frolkis M, Holt SE, Chiu C-P, et al (1998) Extension of Life-Span by Introduction of Telomerase into Normal Human Cells. Science 279: 349–352 doi:10.1126/science.279.5349.349. 9454332 10.1126/science.279.5349.349

[37] Franco S, MacKenzie KL, Dias S, Alvarez S, Rafii S, et al (2001) Clonal Variation in Phenotype and Life Span of Human Embryonic Fibroblasts (MRC-5) Transduced with the Catalytic Component of Telomerase (hTERT). Experimental Cell Research 268: 14–25 doi:10.1006/excr.2001.5264. 11461114 10.1006/excr.2001.5264

[38] O’Sullivan RJ, Karlseder J (2010) Telomeres: protecting chromosomes against genome instability. Nat Rev Mol Cell Biol 11: 171–181 doi:10.1038/nrm2848. 20125188 10.1038/nrm2848 PMC2842081

[39] Morales CP, Holt SE, Ouellette M, Kaur KJ, Yan Y, et al (1999) Absence of cancer-associated changes in human fibroblasts immortalized with telomerase. Nat Genet 21: 115–118 doi:10.1038/5063. 9916803 10.1038/5063

[40] Smith MC, Bayless AM, Goddard ET, Davido DJ (2011) CK2 inhibitors increase the sensitivity of HSV-1 to interferon-[beta]. Antiviral Research 91: 259–266 doi:16/j.antiviral.2011.06.009. 21722672 10.1016/j.antiviral.2011.06.009 PMC3159797

[41] Mossman KL, Macgregor PF, Rozmus JJ, Goryachev AB, Edwards AM, et al (2001) Herpes Simplex Virus Triggers and Then Disarms a Host Antiviral Response. J Virol 75: 750–758 doi:10.1128/JVI.75.2.750-758.2001. 11134288 10.1128/JVI.75.2.750-758.2001 PMC113971

[42] Samaniego LA, Wu N, DeLuca NA (1997) The herpes simplex virus immediate-early protein ICP0 affects transcription from the viral genome and infected-cell survival in the absence of ICP4 and ICP27. J Virol 71: 4614–4625. 9151855 10.1128/jvi.71.6.4614-4625.1997 PMC191683

[43] Counter CM, Hahn WC, Wei W, Dickinson Caddle S, Beijersbergen RL, et al (1998) Dissociation among in vitro telomerase activity, telomere maintenance, and cellular immortalization. Proceedings of the National Academy of Sciences of the United States of America 95: 14723–14728. 9843956 10.1073/pnas.95.25.14723 PMC24516

[44] Misawa K, Nosaka T, Morita S, Kaneko A, Nakahata T, et al (2000) A Method to Identify cDNAs Based on Localization of Green Fluorescent Protein Fusion Products. PNAS 97: 3062–3066 doi:10.1073/pnas.97.7.3062. 10716735 10.1073/pnas.060489597 PMC16192

[45] Dimmer EC, Huntley RP, Alam-Faruque Y, Sawford T, O’Donovan C, et al (2012) The UniProt-GO Annotation database in 2011. Nucleic Acids Res 40: D565–D570 doi:10.1093/nar/gkr1048. 22123736 10.1093/nar/gkr1048 PMC3245010

[46] Hahn WC, Dessain SK, Brooks MW, King JE, Elenbaas B, et al (2002) Enumeration of the Simian Virus 40 Early Region Elements Necessary for Human Cell Transformation. Mol Cell Biol 22: 2111–2123 doi:10.1128/MCB.22.7.2111-2123.2002. 11884599 10.1128/MCB.22.7.2111-2123.2002 PMC133688

[47] Smith KO (1964) Relationship Between the Envelope and the Infectivity of Herpes Simplex Virus. Proc Soc Exp Biol Med 115: 814–816. 14155835 10.3181/00379727-115-29045

[48] Cai WZ, Schaffer PA (1989) Herpes simplex virus type 1 ICP0 plays a critical role in the de novo synthesis of infectious virus following transfection of viral DNA. J Virol 63: 4579–4589. 2552142 10.1128/jvi.63.11.4579-4589.1989 PMC251091

[49] Schaffer PA, Aron GM, Biswal N, Benyesh-Melnick M (1973) Temperature-sensitive mutants of herpes simplex virus type 1: isolation, complementation and partial characterization. Virology 52: 57–71. 4372782 10.1016/0042-6822(73)90398-x

[50] Davido DJ, Zagorski WF, Lane WS, Schaffer PA (2005) Phosphorylation Site Mutations Affect Herpes Simplex Virus Type 1 ICP0 Function. Journal of Virology 79: 1232–1243 doi:10.1128/JVI.79.2.1232-1243.2005. 15613350 10.1128/JVI.79.2.1232-1243.2005 PMC538545

[51] Das SC, Nayak D, Zhou Y, Pattnaik AK (2006) Visualization of Intracellular Transport of Vesicular Stomatitis Virus Nucleocapsids in Living Cells. J Virol 80: 6368–6377 doi:10.1128/JVI.00211-06. 16775325 10.1128/JVI.00211-06 PMC1488946

[52] Van der Loo B, Fenton MJ, Erusalimsky JD (1998) Cytochemical Detection of a Senescence-Associated β-Galactosidase in Endothelial and Smooth Muscle Cells from Human and Rabbit Blood Vessels. Experimental Cell Research 241: 309–315 doi:10.1006/excr.1998.4035. 9637772 10.1006/excr.1998.4035

[53] Hou M, Xu D, Björkholm M, Gruber A (2001) Real-Time Quantitative Telomeric Repeat Amplification Protocol Assay for the Detection of Telomerase Activity. Clinical Chemistry 47: 519–524. 11238306

[54] Zhu H, Zheng C, Xing J, Wang S, Li S, et al (2011) Varicella-Zoster Virus Immediate-Early Protein ORF61 Abrogates the IRF3-Mediated Innate Immune Response through Degradation of Activated IRF3. Journal of Virology 85: 11079–11089 doi:10.1128/JVI.05098-11. 21835786 10.1128/JVI.05098-11 PMC3194975

[55] Hardy GAD, Sieg SF, Rodriguez B, Jiang W, Asaad R, et al (2009) Desensitization to type I interferon in HIV-1 infection correlates with markers of immune activation and disease progression. Blood 113: 5497–5505 doi:10.1182/blood-2008-11-190231. 19299650 10.1182/blood-2008-11-190231 PMC2689050

[56] Kuo R-L, Zhao C, Malur M, Krug RM (2010) Influenza A virus strains that circulate in humans differ in the ability of their NS1 proteins to block the activation of IRF3 and interferon-β transcription. Virology 408: 146–158 doi:10.1016/j.virol.2010.09.012. 20934196 10.1016/j.virol.2010.09.012 PMC2975781

[57] Eidson KM, Hobbs WE, Manning BJ, Carlson P, DeLuca NA (2002) Expression of Herpes Simplex Virus ICP0 Inhibits the Induction of Interferon-Stimulated Genes by Viral Infection. J Virol 76: 2180–2191 doi:10.1128/jvi.76.5.2180-2191.2002. 11836395 10.1128/jvi.76.5.2180-2191.2002 PMC153810

[58] ATCC: Catalog Search (n.d.). Available:http://www.atcc.org/ATCCAdvancedCatalogSearch/ProductDetails/tabid/452/Default.aspx?ATCCNum=CCL-137&Template=cellBiology. Accessed 26 November 2012.

[59] Kim NW, Piatyszek MA, Prowse KR, Harley CB, West MD, et al (1994) Specific Association of Human Telomerase Activity with Immortal Cells and Cancer. Science 266: 2011–2015. 7605428 10.1126/science.7605428

[60] Klingelhutz AJ, Foster SA, McDougall JK (1996) Telomerase activation by the E6 gene product of human papillomavirus type 16. Nature 380: 79–82 doi:10.1038/380079a0. 8598912 10.1038/380079a0

[61] Chen QM, Tu VC, Catania J, Burton M, Toussaint O, et al (2000) Involvement of Rb family proteins, focal adhesion proteins and protein synthesis in senescent morphogenesis induced by hydrogen peroxide. Journal of cell science 113: 4087–4097. 11058095 10.1242/jcs.113.22.4087

[62] Dimri GP, Lee X, Basile G, Acosta M, Scott G, et al (1995) A biomarker that identifies senescent human cells in culture and in aging skin in vivo. Proceedings of the National Academy of Sciences of the United States of America 92: 9363–9367 doi:VL-92. 7568133 10.1073/pnas.92.20.9363 PMC40985

[63] Brailovsky CA, Berman LD, Chany C (1969) Decreased interferon sensitivity and production in cells transformed by SV40 and other oncogenic agents. Int J Cancer 4: 194–203. 4310453 10.1002/ijc.2910040209

[64] Fensterl V, Sen GC (2011) The ISG56/IFIT1 Gene Family. Journal of Interferon & Cytokine Research 31: 71–78 doi:10.1089/jir.2010.0101. 20950130 10.1089/jir.2010.0101 PMC3021354

[65] Rathi AV, Cantalupo PG, Sarkar SN, Pipas JM (2010) Induction of interferon-stimulated genes by Simian virus 40 T antigens. Virology 406: 202–211 doi:10.1016/j.virol.2010.07.018. 20692676 10.1016/j.virol.2010.07.018 PMC2939315

[66] Mossman KL, Saffran HA, Smiley JR (2000) Herpes Simplex Virus ICP0 Mutants Are Hypersensitive to Interferon. J Virol 74: 2052–2056 doi:10.1128/JVI.74.4.2052-2056.2000. 10644380 10.1128/jvi.74.4.2052-2056.2000 PMC111685

[67] Ito Y, Montagnier L (1977) Heterogeneity of the sensitivity of vesicular stomatitis virus to interferons. Infection and immunity 18: 23–27. 198374 10.1128/iai.18.1.23-27.1977 PMC421187

[68] Stewart WE, Scott WD, Sulkin SE (1969) Relative Sensitivities of Viruses to Different Species of Interferon. J Virol 4: 147–153. 4308914 10.1128/jvi.4.2.147-153.1969 PMC375849

[69] Taylor RT, Bresnahan WA (2005) Human Cytomegalovirus Immediate-Early 2 Gene Expression Blocks Virus-Induced Beta Interferon Production. J Virol 79: 3873–3877 doi:10.1128/JVI.79.6.3873-3877.2005. 15731283 10.1128/JVI.79.6.3873-3877.2005 PMC1075717

[70] Langford MP, Weigent DA, Stanton GJ, Baron S (1981) Virus plaque-reduction assay for interferon: Microplaque and regular macroplaque reduction assays. In: Academic Press, Vol. Volume Sidney Pestka, editor. Methods in Enzymology. 78: 339–346. 10.1016/0076-6879(81)78139-4 6173610

[71] Collins SE, Noyce RS, Mossman KL (2004) Innate Cellular Response to Virus Particle Entry Requires IRF3 but Not Virus Replication. J Virol 78: 1706–1717 doi:10.1128/JVI.78.4.1706-1717.2004. 14747536 10.1128/JVI.78.4.1706-1717.2004 PMC369475

[72] Mossman KL, Smiley JR (2002) Herpes Simplex Virus ICP0 and ICP34.5 Counteract Distinct Interferon-Induced Barriers to Virus Replication. J Virol 76: 1995–1998 doi:10.1128/JVI.76.4.1995-1998.2002. 11799195 10.1128/JVI.76.4.1995-1998.2002 PMC135894

[73] Norman KL, Farassati F, Lee PWK (2001) Oncolytic viruses and cancer therapy. Cytokine & Growth Factor Reviews 12: 271–282 doi:10.1016/S1359-6101(00)00024-1. 11325607 10.1016/s1359-6101(00)00024-1

[74] Barber GN (2005) VSV-tumor selective replication and protein translation. Oncogene 24: 7710–7719 doi:10.1038/sj.onc.1209042. 16299531 10.1038/sj.onc.1209042

[75] Cai WZ, Schaffer PA (1991) A cellular function can enhance gene expression and plating efficiency of a mutant defective in the gene for ICP0, a transactivating protein of herpes simplex virus type 1. J Virol 65: 4078–4090. 1649316 10.1128/jvi.65.8.4078-4090.1991 PMC248840

[76] Douglas JL, Quinlan MP (1995) Efficient nuclear localization and immortalizing ability, two functions dependent on the adenovirus type 5 (Ad5) E1A second exon, are necessary for cotransformation with Ad5 E1B but not with T24ras. J Virol 69: 8061–8065. 7494322 10.1128/jvi.69.12.8061-8065.1995 PMC189754

[77] Bartek J, Bartkova J, Kyprianou N, Lalani EN, Staskova Z, et al (1991) Efficient immortalization of luminal epithelial cells from human mammary gland by introduction of simian virus 40 large tumor antigen with a recombinant retrovirus. Proceedings of the National Academy of Sciences 88: 3520. 10.1073/pnas.88.9.3520 PMC51483 1708884

[78] Wazer DE, Liu XL, Chu Q, Gao Q, Band V (1995) Immortalization of Distinct Human Mammary Epithelial Cell Types by Human Papilloma Virus 16 E6 or E7. PNAS 92: 3687–3691. 7537374 10.1073/pnas.92.9.3687 PMC42026

[79] Shay JW, Wright WE, Werbin H (1991) Defining the molecular mechanisms of human cell immortalization. Biochimica et Biophysica Acta (BBA) - Reviews on Cancer 1072: 1–7 doi:10.1016/0304-419X(91)90003-4. 1850299 10.1016/0304-419x(91)90003-4

[80] Ahuja D, S|[aacute]|enz-Robles MT, Pipas JM (2005) SV40 large T antigen targets multiple cellular pathways to elicit cellular transformation. Oncogene 24: 7729–7745 doi:10.1038/sj.onc.1209046. 16299533 10.1038/sj.onc.1209046

[81] Brehm A, Nielsen SJ, Miska EA, McCance DJ, Reid JL, et al (1999) The E7 oncoprotein associates with Mi2 and histone deacetylase activity to promote cell growth. The EMBO Journal 18: 2449–2458 doi:10.1093/emboj/18.9.2449. 10228159 10.1093/emboj/18.9.2449 PMC1171327

[82] Patel D, Huang S-M, Baglia LA, McCance DJ (1999) The E6 protein of human papillomavirus type 16 binds to and inhibits co-activation by CBP and p300. The EMBO Journal 18: 5061–5072 doi:10.1093/emboj/18.18.5061. 10487758 10.1093/emboj/18.18.5061 PMC1171577

[83] Ali SH, DeCaprio JA (2001) Cellular transformation by SV40 large T antigen: interaction with host proteins. Seminars in Cancer Biology 11: 15–23 doi:10.1006/scbi.2000.0342. 11243895 10.1006/scbi.2000.0342

[84] Valls E, De la Cruz X, Martínez-Balbás MA (2003) The SV40 T antigen modulates CBP histone acetyltransferase activity. Nucleic Acids Res 31: 3114–3122. 12799439 10.1093/nar/gkg418 PMC162251

[85] Frisch SM, Mymryk JS (2002) Adenovirus-5 E1A: paradox and paradigm. Nature Reviews Molecular Cell Biology 3: 441–452 doi:10.1038/nrm827. 12042766 10.1038/nrm827

[86] Brockmann D, Esche H (2003) The multifunctional role of E1A in the transcriptional regulation of CREB/CBP-dependent target genes. Curr Top Microbiol Immunol 272: 97–129. 12747548 10.1007/978-3-662-05597-7_4

[87] Hyland PL, McDade SS, McCloskey R, Dickson GJ, Arthur K, et al. (2011) Evidence for Alteration of EZH2, BMI1 and KDM6A and Epigenetic Reprogramming in Human Papillomavirus Type-16 E6/E7 Expressing Keratinocytes. J Virol. Available:http://jvi.asm.org/content/early/2011/08/24/JVI.00160-11. Accessed 2012 June 18. 10.1128/JVI.00160-11 PMC3194988 21865393

[88] Li HP, Leu YW, Chang YS (2005) Epigenetic changes in virus-associated human cancers. Cell Research 15: 262–271 doi:10.1038/sj.cr.7290295. 15857581 10.1038/sj.cr.7290295

[89] Cantalupo PG, Sáenz-Robles MT, Rathi AV, Beerman RW, Patterson WH, et al (2009) Cell-type specific regulation of gene expression by simian virus 40 T antigens. Virology 386: 183–191 doi:10.1016/j.virol.2008.12.038. 19201438 10.1016/j.virol.2008.12.038 PMC2737743

[90] Zhang JJ, Vinkemeier U, Gu W, Chakravarti D, Horvath CM, et al (1996) Two Contact Regions Between Stat1 and CBP/P300 in Interferon Γ Signaling. PNAS 93: 15092–15096. 8986769 10.1073/pnas.93.26.15092 PMC26361

[91] Ronco LV, Karpova AY, Vidal M, Howley PM (1998) Human papillomavirus 16 E6 oncoprotein binds to interferon regulatory factor-3 and inhibits its transcriptional activity. Genes Dev 12: 2061–2072 doi:10.1101/gad.12.13.2061. 9649509 10.1101/gad.12.13.2061 PMC316980

[92] Sundararajan R, White E (2001) E1B 19K Blocks Bax Oligomerization and Tumor Necrosis Factor Alpha-Mediated Apoptosis. J Virol 75: 7506–7516 doi:10.1128/JVI.75.16.7506-7516.2001. 11462023 10.1128/JVI.75.16.7506-7516.2001 PMC114986

[93] Han J, Sabbatini P, Perez D, Rao L, Modha D, et al (1996) The E1B 19K Protein Blocks Apoptosis by Interacting with and Inhibiting the P53-Inducible and Death-Promoting Bax Protein. Genes Dev 10: 461–477 doi:10.1101/gad.10.4.461. 8600029 10.1101/gad.10.4.461

[94] Debbas M, White E (1993) Wild-Type P53 Mediates Apoptosis by E1A, Which Is Inhibited by E1B. Genes Dev 7: 546–554 doi:10.1101/gad.7.4.546. 8384580 10.1101/gad.7.4.546

[95] Rajan P, Swaminathan S, Zhu J, Cole CN, Barber G, et al (1995) A novel translational regulation function for the simian virus 40 large-T antigen gene. J Virol 69: 785–795. 7815544 10.1128/jvi.69.2.785-795.1995 PMC188643

[96] Lindvall C, Hou M, Komurasaki T, Zheng C, Henriksson M, et al (2003) Molecular Characterization of Human Telomerase Reverse Transcriptase-immortalized Human Fibroblasts by Gene Expression Profiling: Activation of the Epiregulin Gene. Cancer Res 63: 1743–1747. 12702554

[97] Bresnahan WA, Hultman GE, Shenk T (2000) Replication of Wild-Type and Mutant Human Cytomegalovirus in Life-Extended Human Diploid Fibroblasts. J Virol 74: 10816–10818 doi:10.1128/JVI.74.22.10816-10818.2000. 11044129 10.1128/jvi.74.22.10816-10818.2000 PMC110959

[98] McSharry BP, Jones CJ, Skinner JW, Kipling D, Wilkinson GWG (2001) Human telomerase reverse transcriptase-immortalized MRC-5 and HCA2 human fibroblasts are fully permissive for human cytomegalovirus. J Gen Virol 82: 855–863. 11257191 10.1099/0022-1317-82-4-855

[99] Xin H, Pereira-Smith OM, Choubey D (2004) Role of IFI 16 in cellular senescence of human fibroblasts. Oncogene 23: 6209–6217 doi:10.1038/sj.onc.1207836. 15208661 10.1038/sj.onc.1207836

[100] Nguyen ML, Kraft RM, Aubert M, Goodwin E, DiMaio D, et al (2007) p53 and hTERT Determine Sensitivity to Viral Apoptosis. J Virol 81: 12985–12995 doi:10.1128/JVI.01485-07. 17855516 10.1128/JVI.01485-07 PMC2169073

[101] Lilley CE, Chaurushiya MS, Boutell C, Everett RD, Weitzman MD (2011) The Intrinsic Antiviral Defense to Incoming HSV-1 Genomes Includes Specific DNA Repair Proteins and Is Counteracted by the Viral Protein ICP0. PLoS Pathog 7: e1002084 doi:10.1371/journal.ppat.1002084. 21698222 10.1371/journal.ppat.1002084 PMC3116817

[102] Halford W, Weisend C, Grace J, Soboleski M, Carr D, et al (2006) ICP0 antagonizes Stat 1-dependent repression of herpes simplex virus: implications for the regulation of viral latency. Virology Journal 3: 44 doi:10.1186/1743-422X-3-44. 16764725 10.1186/1743-422X-3-44 PMC1557838

[103] Kalamvoki M, Roizman B (2009) ICP0 enables and monitors the function of D cyclins in herpes simplex virus 1 infected cells. Proceedings of the National Academy of Sciences 106: 14576–14580 doi:10.1073/pnas.0906905106. 10.1073/pnas.0906905106 PMC2732861 19706544

